# A Systematic Review of the Financial Impact of Living as an Autistic Person in the UK

**DOI:** 10.1192/bjo.2024.77

**Published:** 2024-08-01

**Authors:** Mary Bowley, Mary Jordan

**Affiliations:** University of Warwick Medical School, Coventry, United Kingdom

## Abstract

**Aims:**

Being an autistic person in the United Kingdom (UK) is associated with a range of costs. This study reviews published literature which estimates the cost of living as an autistic person in the UK.

**Methods:**

A systematic review of published peer-reviewed studies was undertaken. Search criteria included papers which were published after 2008, looked exclusively at costs of living as an autistic person in the UK and discussed quantitative data. Papers recovered during the literature search were screened by title and abstract independently by two reviewers. Papers included in the final review were critically appraised using the Critical Appraisal Skills Program (CASP) checklist. Four papers were selected for inclusion in this study. Following data extraction, results were compared in a narrative synthesis across six key domains defined in previous literature: Caregiver costs, Loss of productivity, Healthcare costs, Education costs, Accommodation costs, Therapeutic costs. During the data extraction process, analysis of cost inclusion criteria, data collection methods and cohort characteristics was conducted.

**Results:**

Across the literature the following findings emerged: Costs for autistic people with co-occurring intellectual disability (ID) are higher per year than for those without a co-occurring ID. Costs of care vary with age, with different cost categories peaking at different points in a person's life. Loss of productivity is one of the greatest costs, with education and accommodation costs also proving significant. Data looking at a wide range of expenses however do not determine whether expenses are paid by the individual or by the Government. There is a lack of data regarding financial income, whether sourced from employment or government support, such as Personal Independent Payments or Universal Credit. A lack of consistency regarding cost inclusion criteria and differences in data collection methods severely limit direct comparison of outcomes across the literature.

**Conclusion:**

Lack of consistency in the measurement of cost components and defined cohort characteristics makes comparison across the literature challenging, comparison cannot inform any meaningful economic evaluation. Despite this, the overarching theme across all studies in this review is that current service expenditure is higher for autistic people than non-autistic people. This is particularly clear when discussing accommodation, healthcare and costs due to loss of productivity. Both age and co-occurring conditions have an impact on overall cost. These findings form a strong basis for future research in this area to standardise cost calculations across specified age ranges and evaluate current government-centered financial support available to autistic people.

